# Seeking help for mental health during the COVID-19 pandemic: A longitudinal analysis of adults’ experiences with digital technologies and services

**DOI:** 10.1371/journal.pdig.0000402

**Published:** 2023-12-06

**Authors:** Christine E. Parsons, Kirstin. L. Purves, Molly R. Davies, Jessica Mundy, Shannon Bristow, Thalia C. Eley, Gerome Breen, Colette R. Hirsch, Katherine S. Young

**Affiliations:** 1 Department of Clinical Medicine, Interacting Minds Center, Aarhus University, Aarhus, Denmark; 2 Social, Genetic and Developmental Psychiatry Centre, Institute of Psychiatry, Psychology & Neuroscience, King’s College London, London, United Kingdom; 3 NIHR Maudsley Biomedical Research Centre, King’s College London, United Kingdom; 4 Department of Psychology, Institute of Psychiatry, Psychology and Neuroscience, King’s College London, London, United Kingdom; 5 South London and Maudsley NHS Foundation Trust, London, United Kingdom; Iran University of Medical Sciences, IRAN (ISLAMIC REPUBLIC OF)

## Abstract

The COVID-19 pandemic brought about dramatic changes in how patients access healthcare from its outset. Lockdown restrictions and remote working led to a proliferation of digital technologies and services, which also impacted mental health provisions. Against the backdrop of new and changing support services, along with an unprecedented emphasis on mental health, relatively little is known about how adults sought out and received support for their mental health during this period. With a sample of 27,574 adults assessed longitudinally online over 12 months of the pandemic in the UK, we analysed reports of help-seeking for mental health, as well as sources of treatment or support and the perceived helpfulness of treatments received. We observed that the proportions of participants who reported seeking help remained relatively consistent throughout the 12-month period (ranging from 12.6% to 17.0%). Online talking therapies were among the most frequently sought sources (15.3%), whereas online self-guided treatments were among the least frequently sought sources (5%). Telephone lines, both NHS and non–governmental, had marked treatment ‘gaps’. These treatment gaps, where individuals sought treatment but did not receive it, were especially evident for men and older adults. Our findings underscore online talking therapies as being a widely-sought and helpful source of mental health support. This is important given the current global need for accessible treatment options.

## Author summary

The COVID-19 pandemic and the resulting lockdowns brought about rapid changes in access to healthcare. For mental health, many digital services and technologies were developed and launched. Policymakers, healthcare professionals, the media and the public were deeply concerned about a mental health crisis. However, we know little about how adults sought help for their mental health during the pandemic generally. We used a large cohort of volunteers in the UK to assess the types of treatment that adults sought for their mental health over 12 months. We asked adults if they received help when they sought it, and if the help they received was useful. Over our study period, we found little variation in the number of adults reporting help seeking each month, suggesting no large changes associated with lockdowns or lifting of restrictions. After general practitioners (GPs) and existing mental health teams, online talking therapy was the most sought form of support. Adults who sought online talking therapies reported, on average, that they found them to be very or extremely helpful. However, self-guided treatments, treatments designed to be delivered without extra human support, were among the least popular sources sought. For adults who sought help from telephone lines, many said that they did not receive help, particularly men and older adults. Our data shows that online talking therapies are a widely-sought and helpful source of mental health support, of importance given the worldwide need for accessible mental health treatment.

## Introduction

From the earliest days of the first pandemic lockdown in the UK and globally, the common mantra was to ‘flatten the curve’ to ensure healthcare systems could function within their capacities [1]. Patients were encouraged to avoid unnecessary face-to-face appointments and many routine healthcare visits were initially cancelled or postponed. Against this backdrop of pressed healthcare systems, there have been concerns about the impact of pandemic restrictions and the pandemic itself on population mental health [2]. Was there an increase in help-seeking for mental health issues? Was access to mental health treatment disrupted, given the prioritization of physical health treatment and reductions of in-person and non-essential services?

Several early studies reported disruptions to mental health services and appointment attendance worldwide. For example, a survey from the WHO has indicated severe COVID-related disruption to mental health services [3]. In the 130 countries included, 93% reported disruptions to one or more of their services for mental, neurological, and substance use disorders, with outpatient and community-based services especially affected. Studies examining expected versus actual appointments for mental health services have also shown reductions in service use. In Brazil, 28% fewer outpatient appointments in mental health were observed, compared to what was expected based on prior records, between March and August 2020 [4]. In the UK, a population-based cohort study reported reductions in primary care-recorded mental illness for adults in April 2020 relative to expected rates [5]. Reductions in recorded illnesses, interpreted as reduced help-seeking from primary care, were particularly pronounced in patients registered with General Practitioners (GPs) in more socially deprived areas.

Health systems worldwide moved quickly to adopt virtual treatment approaches to reduce the need for physical meetings between patients and healthcare providers [6]. For mental health, telepsychiatry, where psychiatric services can be provided through or with the support of technology, had a compelling case for its broader adoption [7]. Beyond telepsychiatry, numerous online services and resources were made available to increase the availability of self-help support (e.g., UCLA’s STAND together programme; https://standtogether.ucla.edu/). In the UK, National Health Service (NHS) mental health crisis telephone helplines were rolled out four years earlier than planned and reached countrywide coverage by May 2020 [8]. The NHS also published numerous psychoeducational websites for managing mental health (e.g., Every Mind Matters [9]) and offered tools such as “Mind Plan [10]” to provide personalised, targeted action plans, with NHS-endorsed advice for improving mood and wellbeing.

The development of virtual care and digital tools for mental health accelerated during the pandemic, given the need for remote, accessible, and scalable support (for reviews, see [11,12]). However, evidence from early in the pandemic indicates that patient uptake of remote consultations, as an example, differed considerably across countries [3]. Low-income countries had particularly low rates of adoption, partly due to low resources. Furthermore, US data showed that patient uptake of remote consultations tended to be lower for specific demographic groups, such as those of non-White ethnicity and older adults [13]. The digital divide is also an important consideration for automated or self-guided interventions, interventions which do not include a human support element. Many self-guided interventions are targeted towards the majority identity population and are not designed with diversity or inclusion in mind [14] and marginalised or low-resource communities often have unique mental health needs [15].

Understanding how people sought and accessed help for their mental health is important, given the large shifts in the types of care and support made available during COVID-19. Along with an unprecedented media focus on mental health and more time at home, it is possible that adults sought mental health help that they had been delaying. Conversely, it is possible that adults sought out help less often, given the emphasis on reducing healthcare system burden. Using data from two longitudinal cohorts in the UK, we examined how adults sought help for mental health issues over a 12-month period from July 2020 to June 2021. Our dataset allowed us to examine if there was an intensification of mental health support-seeking timed around national lockdowns compared to periods with fewer restrictions. We were also able to examine the types of support sought, along with reasons for seeking, whether support was received or not, and demographic predictors of help seeking. In instances where support was sought but not received, we also analysed the reasons participants provided for its non-receipt.

## Method

### Study design

We combined data from two cohorts, the COVID-19 Psychiatry and Neurological Genetics (COPING) study and the Repeated Assessment of Mental Health in Pandemics (RAMP) study. Full details of the COPING and RAMP study procedures are reported elsewhere [16]. All participants were UK residents aged 16 years or above. COPING recruited from existing participant cohorts via the National Institute for Health and Care Research BioResource, including a large proportion of individuals from the Genetic Links to Anxiety and Depression (GLAD) Study [17]. There were a number of methods for initial recruitment to the NIHR BioResource, including through blood donation centres and other National Health Service sites. RAMP recruited via social media advertising (e.g., Facebook, Twitter), the King’s College University website, and word-of-mouth. Table 1 presents demographic data for the participants including age, gender, ethnicity/race, and prior mental health diagnoses. Both cohorts had far higher than population average representation of individuals with prior mental health diagnoses, primarily anxiety and depression.

**Table 1:** Demographic details of the RAMP and COPING cohorts. All data is self-reported, including prior mental health diagnoses.

	COPING (N = 22 068)		RAMP (N = 5 506)		Combined (N = 27 574)	
	N	%	N	%	N	%
**Age (years)**						
16–25	1191	5.40%	567	10.30%	1758	6.38%
26–35	2611	11.83%	563	10.23%	3174	11.51%
36–45	3137	14.22%	513	9.32%	3650	13.24%
46–55	4773	21.63%	1061	19.27%	5834	21.16%
56–65	5555	25.17%	1627	29.55%	7182	26.05%
66–75	3594	16.29%	1015	18.43%	4609	16.72%
76+	305	1.38%	160	2.91%	465	1.69%
Missing	902	4.09%	0	0.00%	902	3.27%
**Gender**						
Male	6846	31.02%	1118	20.31%	7964	28.88%
Female	14873	67.40%	4317	78.41%	19190	69.59%
Non-binary/prefer to self-define	251	1.14%	48	0.87%	299	1.08%
Missing	98	0.44%	23	0.42%	121	0.44%
**Ethnicity—clustered**						
White	18441	83.56%	5212	94.66%	23653	85.78%
Racialised minorities	608	2.76%	271	4.92%	879	3.19%
Missing	3019	13.68%	23	0.42%	3042	11.03%
**Pre-pandemic mental health diagnoses**						
Depressive disorder	11217	50.83%	2711	49.24%	13928	50.51%
Anxiety disorder	9136	41.40%	3247	58.97%	12383	44.91%
Obsessive-compulsive related disorder	893	4.05%	236	4.29%	1129	4.09%
Psychotic disorder	392	1.78%	108	1.96%	500	1.81%
Bipolar disorder	755	3.42%	159	2.89%	914	3.31%
Eating Disorder	1058	4.79%	310	5.63%	1368	4.96%
Personality disorder	882	4.00%	172	3.12%	1054	3.82%
Post-traumatic stress disorder	1862	8.44%	453	8.23%	2315	8.40%
Autism spectrum disorder	240	1.09%	103	1.87%	343	1.24%

Online consent was obtained from all participants, and ethical approval was obtained for COPING from the NHS Health Research Authority, South West—Central Bristol Research Ethics Committee (20/SW/0078) and for RAMP, from the Psychiatry, Nursing and Midwifery Research Ethics Committee at King’s College London (HR-19/20-18157). Participants were given a link to the online questionnaire measures, administered via Qualtrics (Provo, UT). For follow-up surveys, RAMP participants were emailed completion reminders and COPING participants received email and text message reminders.

### Data collection

Participants were recruited on a rolling basis from April to September 2020 and answered questions related to their mental health service use from July 2020. The present study includes data from a 12-month period, from July 2020 until July 2021 (N = 27,574; COPING = 22,068, RAMP = 5,506). Given that RAMP and COPING had different schedules of questionnaire timing, we collapsed data into monthly time intervals, to combine data from the two cohorts. Questionnaires were sent at approximately 2-monthly intervals for each cohort, with each cohort receiving questionnaires in alternating months (i.e., August, October, December etc. in COPING and July, September, November etc. in RAMP). Differences in schedules arose because the treatment questionnaire was sent to participants after the study had already been initiated. COPING and RAMP cohorts received slightly different questionnaire batteries due to differences in the main study questions. Treatment questionnaires were added to the shorter assessment in each cohort (see S1 Table for response rates at each time point). No participant attention checks were included in the questionnaires.

### Measures: Demographics and treatment-seeking

Demographic data was collected at each participant’s first assessment point. We include data on participants’ age (7 categories, see Table 1), ethnicity (2 categories: white or racialised minority) and reporting of prior mental health diagnoses. Several variables (age over 75 years, non-binary and self-defining gender categories, and racialised minority groups) were combined due to small sample sizes. Participants ranged in age from 16–76+ years, with the largest group aged 56–65 years (26.05%). The sample was predominantly female (69.59%) and reported White ethnicity (85.78%). The sample included high proportions of individuals reporting a depressive (50.51%) or anxiety (44.91%) disorder.

To measure service use, we co-designed a mental health-specific service access and use questionnaire with the Department for Health and Social Care, UK. Here, we report on data from the instrument, which asked about participants’: (i) treatment/support seeking, (ii) source of the treatment/support sought, (iii) reasons for treatment non-receipt, (iv) perception of helpfulness, (v) reasons for help-seeking or not help seeking. Most items comprised multiple choice options, where participants could select all that applied (see https://osf.io/7p2ek/ for full instrument and measures included). Treatment seeking comprised a binary variable (yes, no), if participants reported seeking help for themselves for their mental health. For treatment helpfulness (iv), participants rated each source/support on a 7-point Likert scale, from extremely unhelpful to extremely helpful, for each source of help accessed. The first baseline measure asked participants about their treatment/support seeking since the beginning of the pandemic. Follow-up measures referred to the preceding month. We did not ascertain the reliability or validity of the questionnaire.

Options for the ‘sources of treatment sought’ measure included 11 categories, each with a clarifying example, where relevant: (i) Emergency mental health service, (ii) Existing mental health support team (e.g. social worker, CPN, or psychiatrist with whom you have had contact previously), (iii) online talking therapy (e.g. one-to-one therapy or group therapy, led by a therapist), (iv) registered GP, (v) NHS 111 (crisis phone line), (vi) non-NHS helpline (e.g. the Samaritans, Mind or other), (vii) NHS or government support or information website, (viii) non-NHS support or information website (e.g. Samaritans, Mind or other), (ix) online self-guided therapy (e.g. online CBT), (x) structured therapeutic activity (e.g. mindfulness or online self-help), (xi) other. In the UK, CPNs are community psychiatric nurses, and the NHS 111 phone line is for urgent care, but not a life-threatening emergency.

### Statistical analyses

Data cleaning and analysis was conducted using R (version 4.1.2, 2021-11-01 [18]) scripts are available at: https://github.com/RAMP-COPING/CovidTreatment. We included all participants who answered the treatment seeking questionnaires at any time point in the 12-month period. Participants who responded to the treatment seeking questionnaires without providing age and gender responses were included in the larger analyses but excluded from the analyses using these two variables. For each time interval, we analysed all available answers to the treatment seeking questionnaire. Where there were missing responses to items, we included the available responses regardless. For example, if participants responded that they sought help via a self-guided treatment, but did not rate the treatment helpfulness, we included their response to the help seeking source item only. In short, no imputation was performed and we did not exclude participants who provided partial responses. We combined the RAMP and COPING cohorts into one sample, for several reasons. First, the two cohorts responded to the same questionnaire items once per month. Second, combining the two cohorts maximised the available data, and we did not have any hypotheses about between-cohort differences. Descriptive statistics, primarily with percentages where appropriate, are presented for our outcome variables. For comparisons based on gender and age categories, we report Pearson’s Chi-squared tests.

## Results

We report the frequency of five outcomes. These were: treatment seeking, treatment receipt (i.e., what proportion of individuals reported seeking a specific treatment and whether they said they received it or not), reasons for treatment seeking or not seeking, reasons for non-receipt, and perceived helpfulness of treatment received.

### Treatment seeking

**Fig 1:**
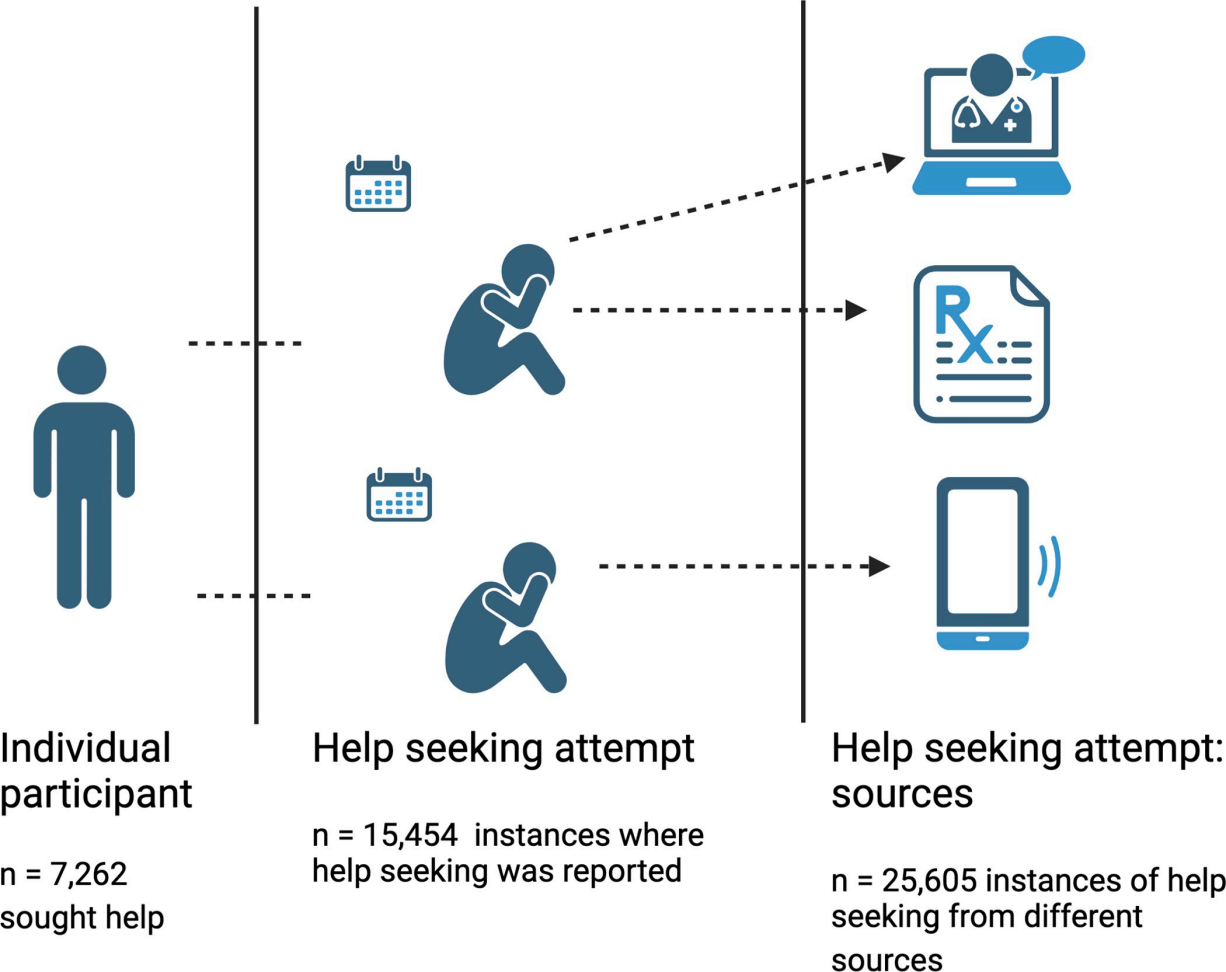
26.33% (7,262) of participants reported seeking help at least once during the 12-month period (July 2020—June 2021).

**Fig 2:**
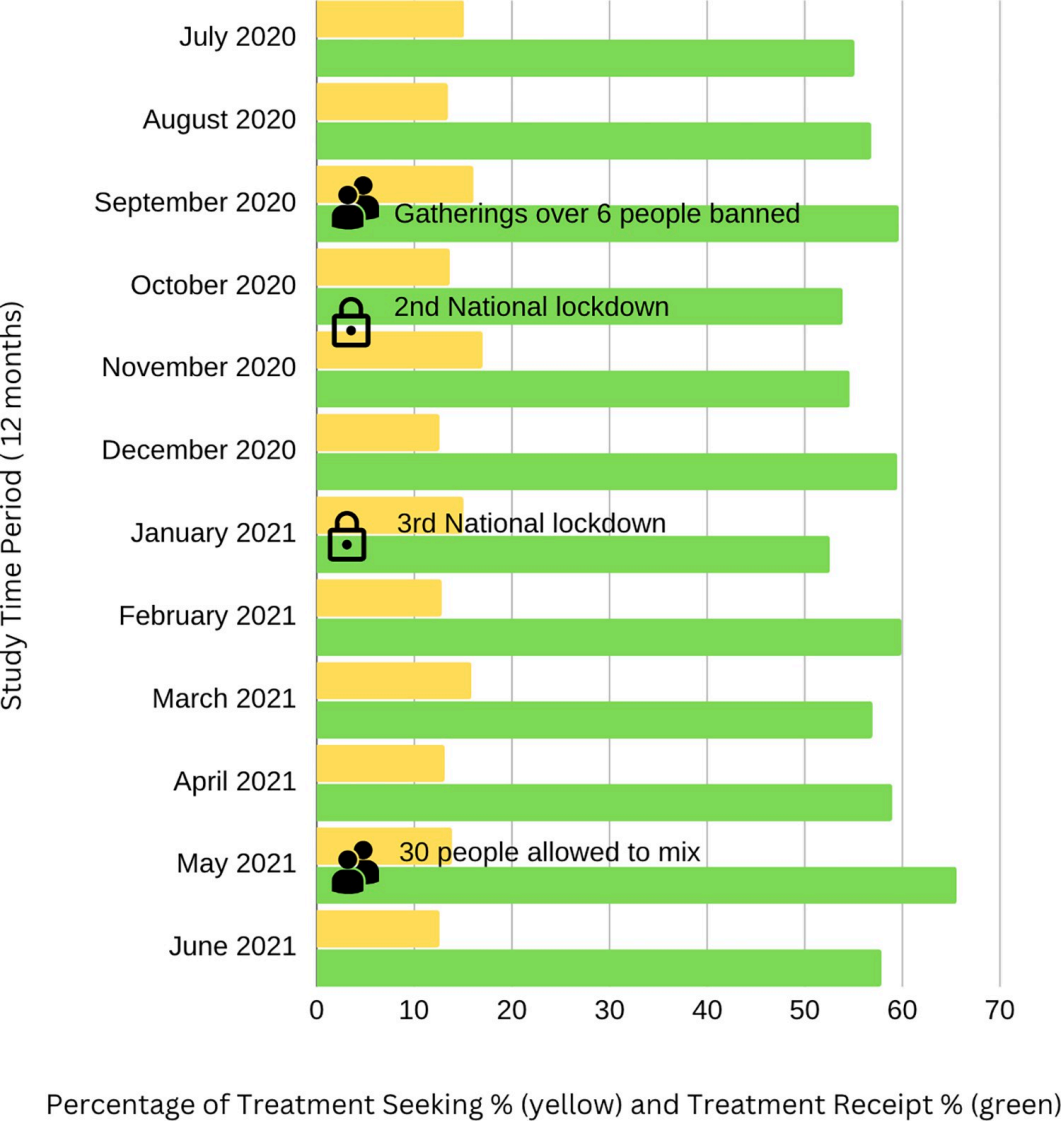
Percentage of treatment seeking and treatment receipt (i.e., the proportion of individuals who reported seeking and receiving treatment). Treatment seeking ranged from 12.6% in June to 17% in November. Treatment receipt ranged between 52.6% in January 2021 and 65.6% in May 2021.

Collapsed across time points, 26.33% of participants (n = 7,262) reported seeking treatment at least once between July 2020 and June 2021. In total, participants reported attempting to seek help from different sources on 25,605 instances (see Fig 1). Each individual could report seeking treatment via multiple sources of treatment during each assessment interval. Over the whole assessment period (July 2020-June 2021), there was a mean of 3.53 treatment seeking attempts per participant who sought help (SD = 4.20). Looking at the distribution of treatment seeking over the total period, of those seeking treatment, 47.40% (3,442) reported seeking treatment during one of the assessed intervals, 23.08% (n = 1,676) during two intervals, 12.81% (n = 930) during three intervals and 16.72% (n = 1,214) during four or more intervals. The proportion of individuals seeking treatment was reasonably consistent across the assessed time points, with 12.6–17.0% reporting treatment seeking across the 12-month study period (see Fig 2).

### Examining rates of seeking of different types of treatment

GPs were the most commonly reported source of help sought by respondents (19.94%). The next most commonly reported sources were either from an existing mental health team (15.90%) or online talking therapy (15.30%; S2 Table). Self-guided online therapies were much less commonly sought compared to online talking therapies, at just 4.96%. Participants also commonly sought help from websites, with government and non-government websites combined comprising 14.18% of help seeking instances. Phone lines were less common sources than websites, with the NHS 111 phone line being the least reported of the eleven options, at 2.17%.

**Fig 3:**
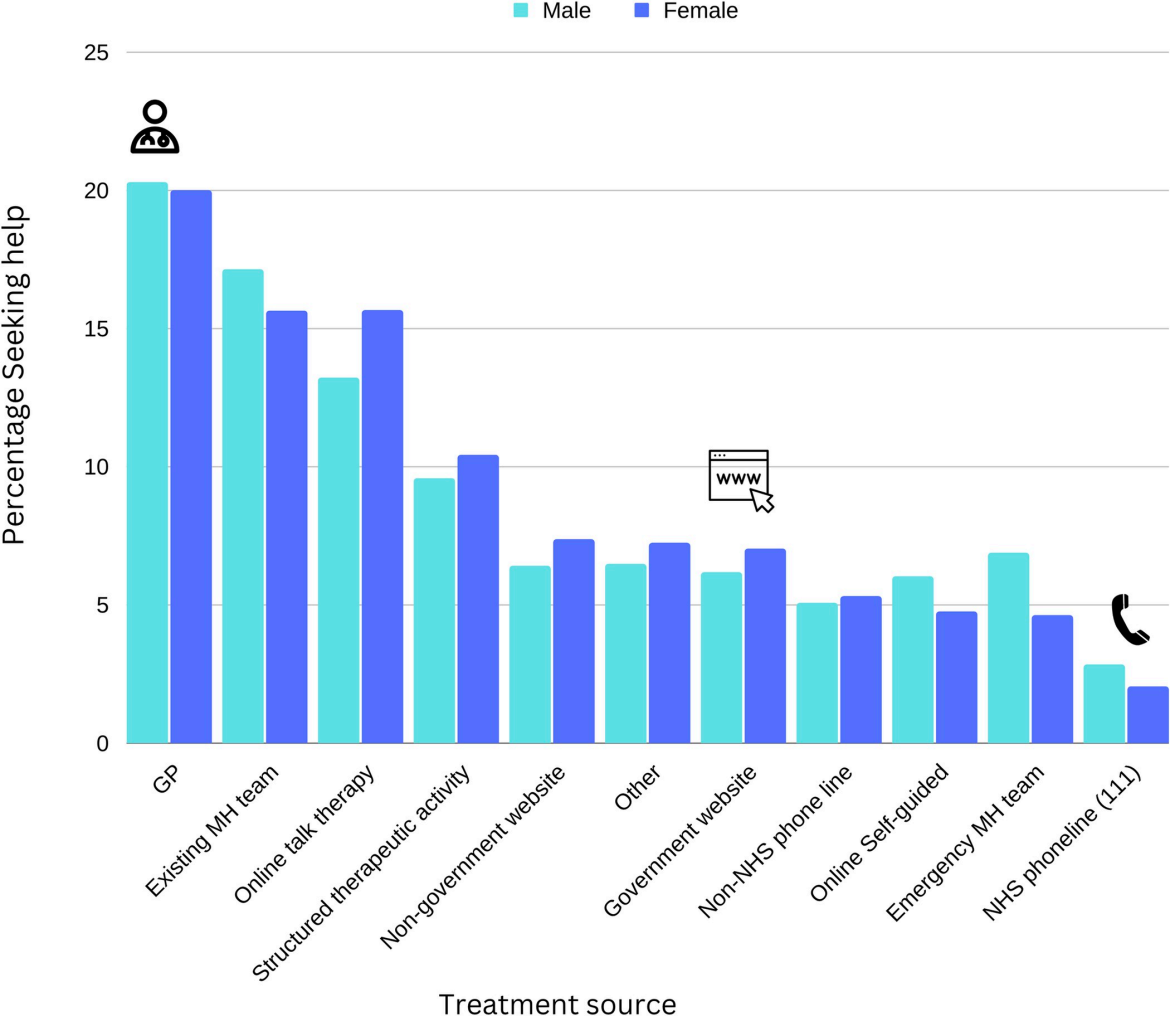
GPs were the most common source of treatment seeking, with high rates for online talking therapies and existing mental health teams as well. The least frequently sought sources were: i) online self-guided support and (ii) NHS phone lines. MH = mental health. NHS = National Health Service. GP = General Practitioner.

When examining gender differences in treatment seeking, we restricted comparisons to men and women because our self-reported non-binary or self-identifying gender group comprised 1% of the total sample size, restricting the statistical power to make valid comparisons with this group. Across all timepoints, frequencies of treatment-seeking were significantly higher among women (16.12%) than men (7.16%, *X*^*2*^ (1, *N* = 112,445) = 1663.9, p = < .001; Fig 3). There were also differences between men and women in terms of the treatment type sought (*X*^*2*^ (10, N = 24,622) = 86.24, *p* < .001). Women reported more frequent seeking of online talking therapy compared to men (15.65% in women vs. 13.21% in men), while men reported higher rates of help seeking from their emergency mental health team compared to women (6.87% in men vs. 4.62% in women; see S3 Table).

**Fig 4:**
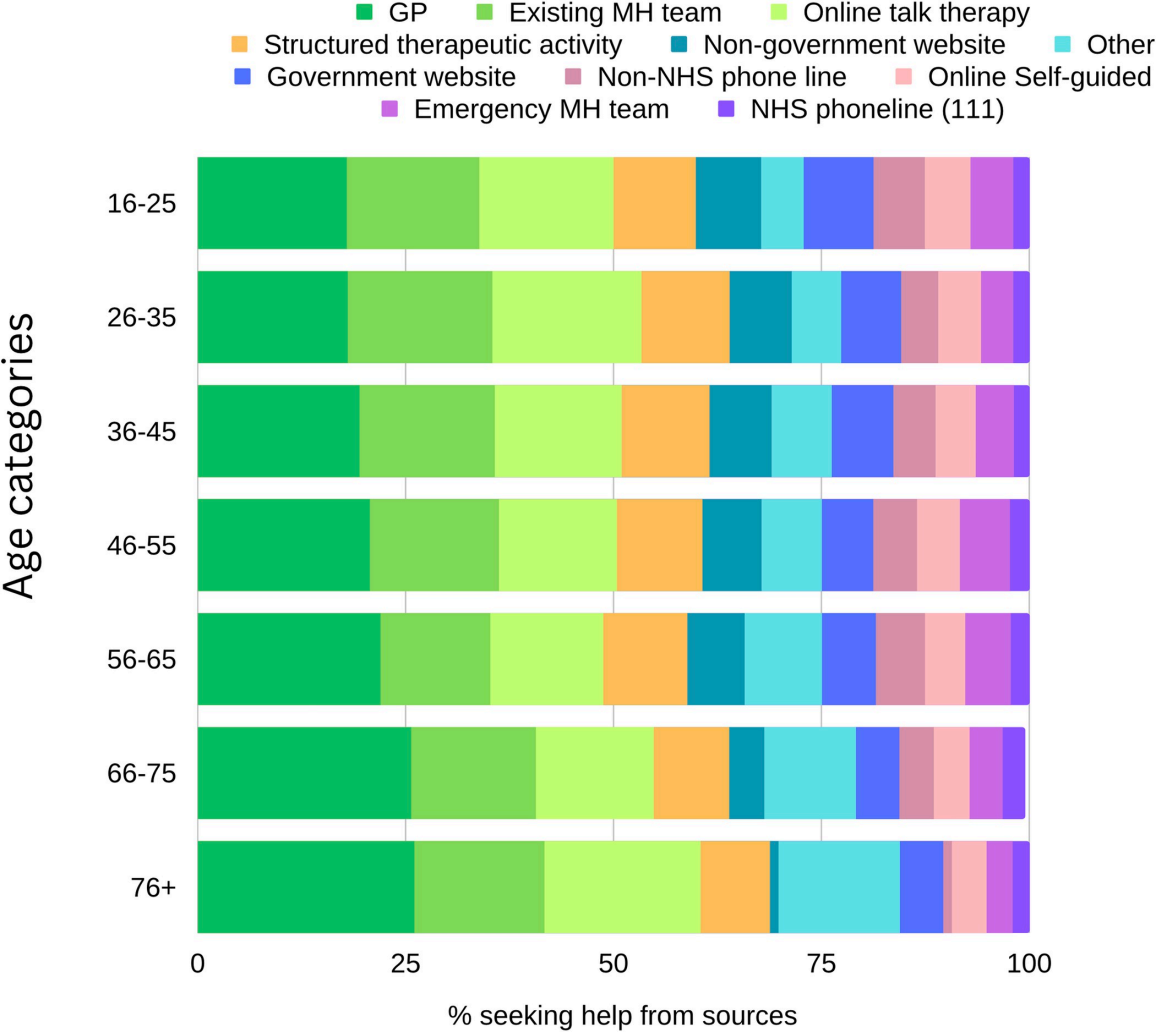
GPs were the most common source of help sought and the rates of help seeking from GPs increased with age.

There was a striking association between age and treatment seeking, with far higher proportions of younger people reporting treatment seeking than older people (*X*^*2*^ (6, *N* = 110996) = 6716.3, *p* = < .001). For types of treatment, a higher proportion of older adults reported seeking help from their GP compared to younger adults (ca. 26% among adults over 66 years, vs. ca. 18% for adults under 35 years). A higher proportion of younger adults reported treatment seeking from non-government websites compared to older adults (Fig 4; S4 Table).

### Treatment receipt

Overall, there were 25,605 instances of treatment seeking, and 14,693 instances in which a specific treatment was sought *and* received (57.38% treatment receipt). We explored treatment receipt across the 12 months of the study, across all treatment sources. We compared the frequency with which individuals sought one or more types of treatment, and where none was received (see Fig 2). The lowest treatment receipt was reported at the time of the third national lockdown in the UK, January 2021 (52.58%) and the highest was recorded in May 2021 (65.56%), around a time of lifting restrictions.

**Fig 5:**
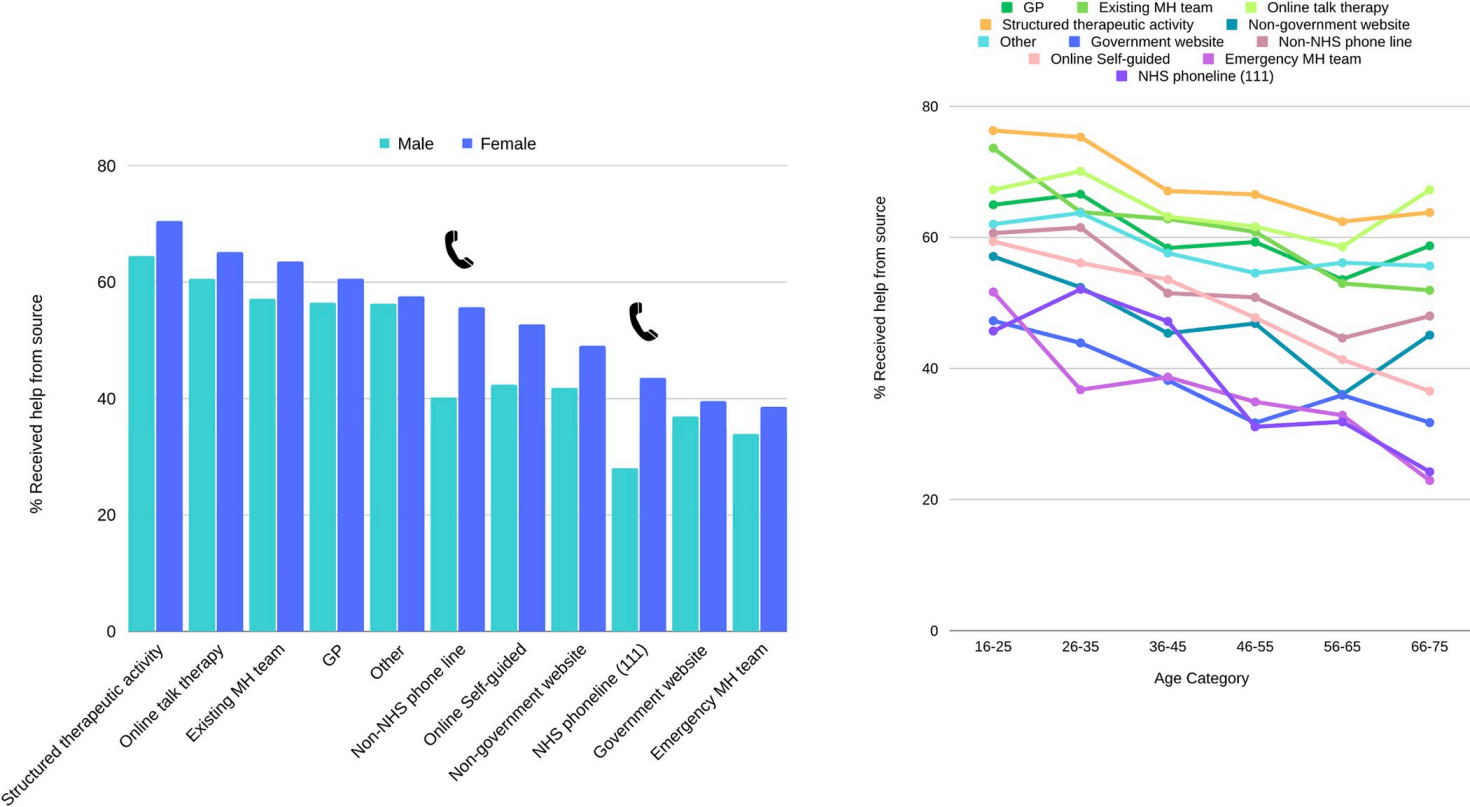
Treatment receipt by source across gender (left) and age (right). Treatment receipt refers to the proportion of individuals who reported seeking and receiving treatment. Overall, women reported higher rates of treatment receipt than men, and younger people reported higher rates of treatment receipt than older people.

Treatment receipt differed widely according to the source of treatment sought (Fig 5). Of concern, only 37.73% of respondents who sought help from an emergency mental health team, and only 39.93% of respondents who sought help from the NHS helpline (111), reported receiving it. The highest rates of treatment receipt were for online talking therapies (64.82%), existing mental health teams (62.74%) and structured therapeutic activities (e.g., mindfulness; 69.56%). Comparing website-based help, participants reported receiving treatment more so from non-government (e.g., third sector organisations) sources (48.33%) than from government (e.g., NHS) sources (39.47%; see S2 Table).

Among treatment seekers, women were more likely to report receiving treatment than men (58.26%, versus 51.74%, *X*^2^ (1, *N* = 24,622) = 58.43, p = < .001). There were particularly pronounced gender differences related to phone lines, where women reported much higher receipt of help from both the NHS and non-NHS phone lines (43.75% and 55.68%, respectively), compared to men (28.07% and 40.20%, respectively). Younger people were more likely to report receiving treatment than older people (63.93% among 16–25-year-olds, 42.71% among 76+ year olds; *X*^*2*^ (6, *N* = 23,783) = 233.55, *p* = < .001; Fig 5; S5 Table). Although older adults reported less treatment receipt across all sources, the difference between younger and older adults (66+ years) in treatment receipt was less pronounced for online talking therapy and for structured therapeutic activities.

### Reasons for seeking or for not seeking treatment

**Fig 6:**
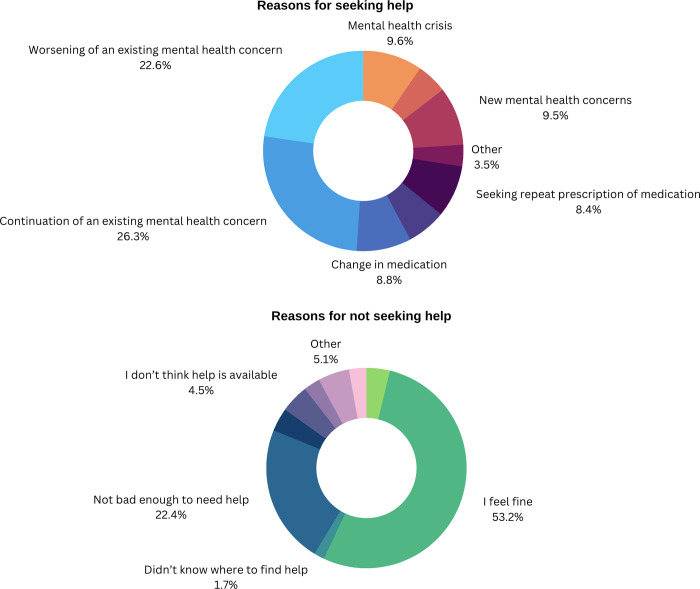
Why did participants seek help for their mental health? The majority reported help seeking for an existing mental health concern that was either continuing or worsening. New mental health concerns made up only a small proportion of overall help seeking instances. For those not seeking help, the main reasons were due to feeling “fine” (53.20%) or “not bad enough to need help” (22.45%).

There was minimal variety in the reasons for treatment-seeking over time across the 12-month study period (see Fig 6; S6 Table). We did not find evidence that stricter physical restrictions were associated with changes in reasons for help-seeking. We did not, for example, see ‘new mental concerns’ as a reason peaking in months where there were physical lockdowns compared to lighter restrictions. Most treatment seeking was related to either a continuation (26.29%) or a worsening of an existing mental health problem (22.65%). Most participants reported that they did not seek treatment because they had no, or only mild symptoms (53.20% selected ‘feel fine’, 22.45% ‘not bad enough to need help’). A smaller group (10.03%) indicated system-oriented reasons (4.55% ‘don’t think help is available’, 3.78% ‘bad past experience’, 1.70% ‘didn’t know where to find help’). Finally, 5.48% of answers indicated reasons related to their own personal feelings or situations (2.67% ‘I want to but haven’t’, 2.81% ‘I’m too busy’).

### Reasons for non-receipt of treatment

**Fig 7:**
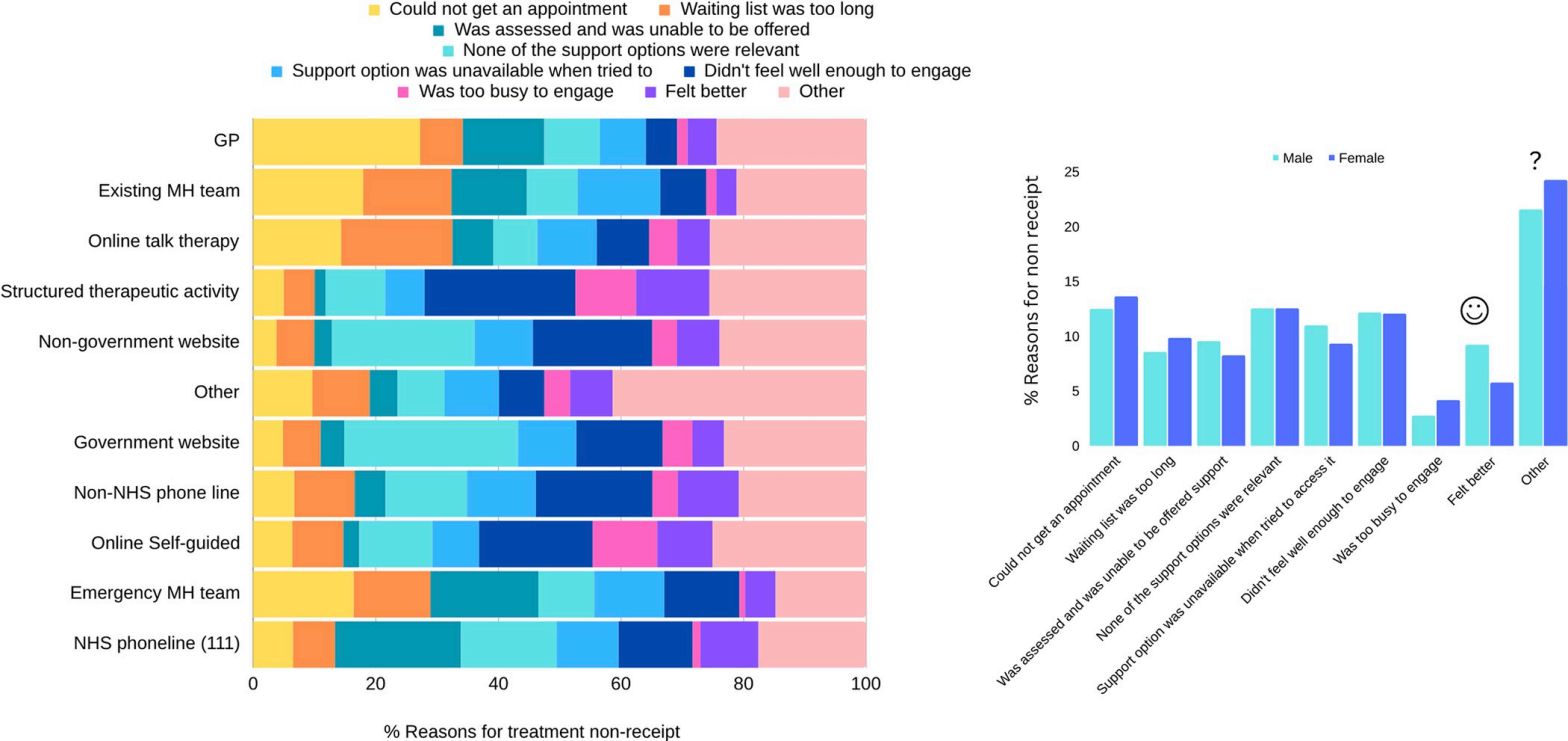
Reasons for treatment non-receipt across treatment type, and for men and women. Among the most frequently sought treatment source (GPs), the most common reason for treatment non-receipt from GPs was not being able to get an appointment. Overall, men and women endorsed each of the provided reasons at similar rates, but men endorsed “felt better” more often than women, and women endorsed “other” more often than men.

For GPs, the most commonly-sought treatment source, the most frequently endorsed reason for non-receipt was appointment unavailability (27.24%; Fig 7, S7 Table). For website-based support, both government and non-government, the most commonly endorsed reason for non-receipt was that the support was not relevant (28.34% and 23.27% respectively). For NHS 111 phone lines, where the largest gap in treatment receipt was reported, “being assessed but being unable to be offered support” was the most common reason (20.52%). There were also significant gender differences in the reasons provided for non-receipt (*X*^*2*^ (8, *N* = 9,938) = 50.64, *p* < .001). Men more often endorsed “felt better” than women (9.24% vs. 5.80%), and women endorsed “other” more often than men (24.27% vs 21.58%; Fig 7, S8 Table).

### Helpfulness of treatment

**Fig 8:**
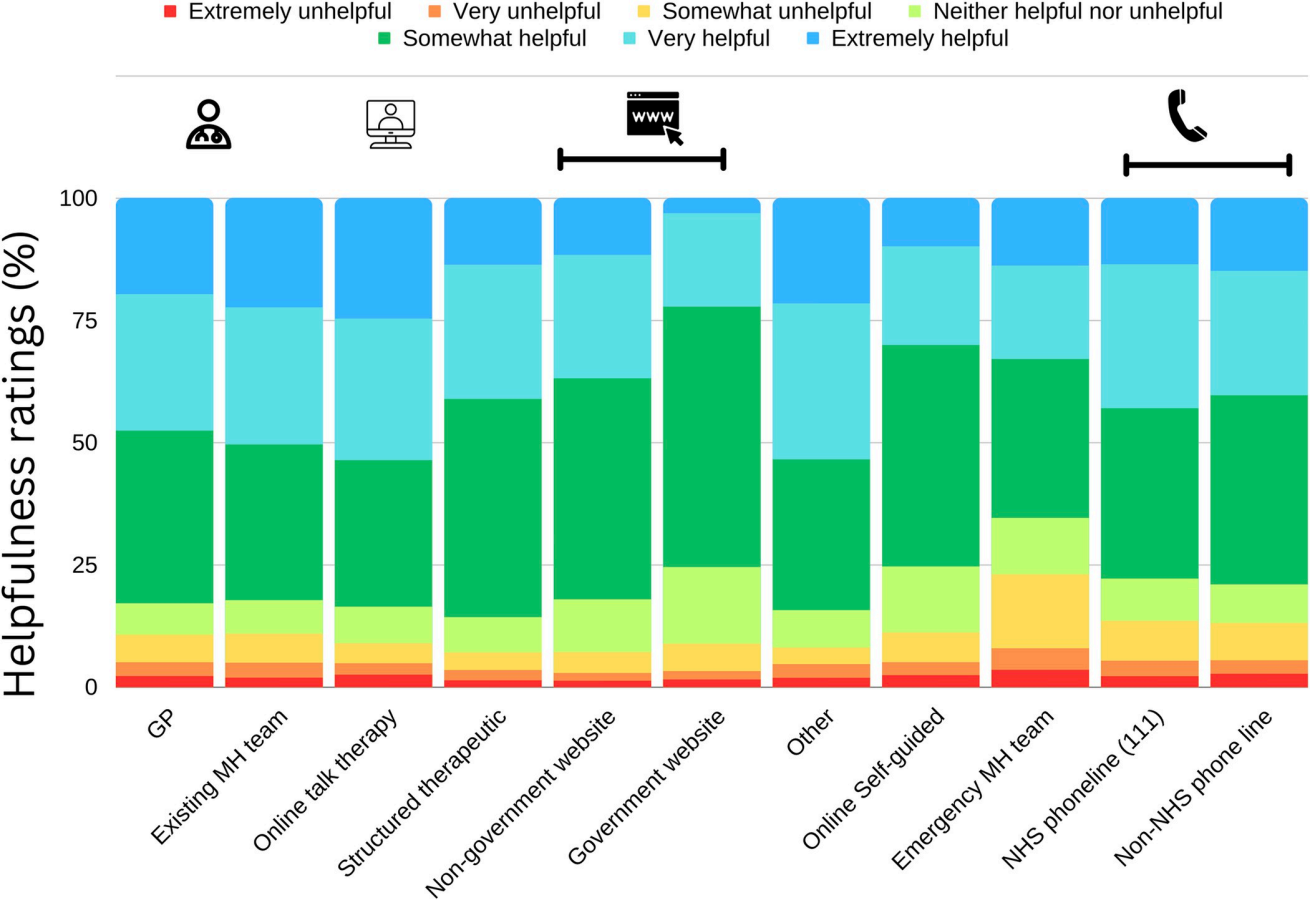
Helpfulness of treatment for each source. Of the two website sources, non-government sites received more positive ratings of helpfulness overall, and online talking therapy was rated more positively compared to online self-guided therapy.

When sought and received, treatment was rated as at least ‘somewhat’ helpful in most instances (81.75%). The most frequent rating was ‘somewhat helpful’ (36.57%), with 27.00% of ratings being ‘very helpful’ and 18.18% of ratings being ‘extremely helpful’. There were fewer ratings of ‘neither helpful nor unhelpful’ (8.14%), somewhat unhelpful (5.38%), very unhelpful (2.60%) or extremely unhelpful (2.13%). Online talking therapies received the highest proportion of ‘extremely helpful’ ratings and were more positively rated compared with self-guided therapies, which had a higher proportion of ‘somewhat helpful’ ratings. For websites, helpfulness ratings were overall more positive for non-government websites compared to government websites (Fig 8; S9 Table).

## Discussion

### COVID-restriction related effects

We did not find evidence for large fluctuations in treatment-seeking for mental health in periods of tighter or more relaxed restrictions in the UK. Treatment seeking in this sample appeared relatively stable across the 12 months from July 2020 onwards, with a 4% difference between the months with the highest reported treatment seeking and the lowest (17% in November 2020, 13% in December 2020). Most individuals were seeking treatment for a continuation or worsening of an existing mental health difficulty. However, we did note larger differences for treatment receipt, with the lowest levels recorded corresponding to the 3rd national lockdown (53%, January 2021), compared to the highest, where social gathering restrictions were eased (66%, May 2021). Whether these small fluctuations were related to COVID restrictions or not cannot be determined by the present data, but our findings are in general alignment with other data sources on treatment-seeking. Appointment record data from General Practitioners (GPs) in England showed slightly lower patient attendance in January 2021 compared to the preceding December or the following February, a difference not seen in January 2022 data (NHS Digital, 2022).

### Online talking therapy is a sought-after treatment

Given that GP consultation is required for accessing most specialist healthcare services in the UK, it is unsurprising that GPs were the most sought form of support, particularly for older adults. After GPs and existing mental health teams, online talking therapy was the most sought form of support. Participants who sought online talking therapies generally reported that they received help, and rates of receipt for online talking therapies were higher than for other sources assessed in our study. Our findings of high treatment receipt of online talking therapies can be considered in terms of general trends in mental health service provision. In the UK, there has been a dramatic increase in internet-enabled therapy sessions delivered as part of the NHS Talking Therapies service (formerly known as Improving Access to Psychological Therapies; IAPT). From September 2020, the NHS Digital libraries started publishing summaries of internet-enabled therapy sessions, and 6,498 sessions were recorded. Just 12 months later, 55,503 sessions were recorded in September 2021 [19].

Previous studies of online talking therapies have usually focused on attrition rates, treatment preferences, and effectiveness, rather than the perceptions of treatment receipt and helpfulness that we investigated. For attrition, internet-delivered guided cognitive behaviour therapy (iCBT) has been shown to have lower overall completion rates compared to face-to-face CBT (67.5% versus 84.7%) prior to COVID-19 [20]. Of note, when we examine non-completers, participants in iCBT formats have been shown to complete more of their treatment than participants in face-to-face formats (42.1% versus 24.5%). For preferences, there is prior evidence that participants prefer face-to-face psychotherapy over online, even though they rate experiences with online therapy as at least ‘good’ [21]. For efficacy, meta-analyses have provided evidence that online modalities can be equivalent to face-to-face. A 2020 analysis, for example, showed that electronically-delivered CBT (web, app, video conferencing) was as effective as face-to-face CBT for depression [22]. Looking more broadly than CBT, an analysis of videoconferencing psychotherapy compared to face-to-face psychotherapy showed non-inferior symptom reduction for the online format [23]. While our measures reflect more global perceptions of online talking therapies (i.e., ‘did you receive help?’), our findings of high treatment receipt broadly align with findings on treatment completion. Over half of our participants who received online talking therapy rated it as ‘very helpful’ or ‘extremely helpful’, consistent with the overall findings on symptom reduction in online therapies.

### Self-guided online therapies are less popular

Although online self-guided therapies have also seen substantial development, they were far less sought after by our participants. In fact, only the NHS 111 phone line was sought less frequently than online self-guided therapy. We also note two issues with participants’ experiences of self-guided online therapies relative to online talking therapies. First, participants who sought help from self-guided online therapies were less likely to report receiving treatment. This effect differed by age. Young adults reported higher rates of receiving treatment from self-guided online therapies compared to older adults, but this pattern was not observed with online talking therapies. Second, individuals who received self-guided treatment reported lower ratings of its helpfulness, relative to online talking therapies.

That self-guided online therapies were relatively unpopular among our participants is surprising, given the growing availability, market development and potential ease of access to options such as apps and chatbots. Despite the increasing evidence that self-guided online therapies are effective [24,25], patient preference for traditional talking therapies remains a barrier to wider uptake of these services. An advantage of self-guided or autonomous therapies is their potentially low financial cost and greater scalability relative to therapist-guided treatments, but more needs to be done to enhance participant engagement [26].

### Gender and age effects

Consistent with a large body of evidence [e.g., 27,28], more women reported seeking help for their mental health than men, and more women reported receiving the help they sought. We found especially pronounced gender differences in treatment receipt related to the two telephone support sources: both NHS 111 phone lines and non-government phone lines. Our finding that men reported relatively low levels of treatment receipt from phone lines is not unexpected given prior work. A recent qualitative study on men’s use of crisis phone lines highlighted that some men have a limited understanding of the type and level of support that helplines can feasibly offer [29]. Given the present findings, providing information on the purpose and scope of telephone lines, particularly for men but not limited to them, may help align expectations with the reality of what phone lines can offer. Older adults (55+ years) also reported low levels of treatment receipt from NHS phone lines, with only a minority reporting that they received treatment. Future research could usefully focus on understanding misalignments of expectations among older adults and how to improve experiences for this group.

The COVID pandemic has had a particular toll on young people’s mental health [16,30,31]. However, young people also seem to be more comfortable discussing their mental health compared to older generations [32], and our results indicate that they seek help for their mental health in higher proportions relative to older adults. Whether young people’s greater treatment seeking is due to a higher symptom burden, or a greater openness to identifying and addressing mental health issues, is an open question. Striking in our data, however, was the near linear decrease in treatment seeking reported with age. Similar findings have been reported in Canada and Australia, showing that older adults are less likely than younger adults to seek help for their mental health [33,34]. Reasons why older adults may not seek help include beliefs that symptoms are a normal part of ageing, a preference for self-reliance, concerns about the cost of treatment, or fears about taking medication [34].

### Implications for clinical practice, policy and for technology developers

We highlight several findings that may inform clinical practice and online treatment development. For technology developers, our data suggests that improving the marketing and appeal of self-guided therapies could be beneficial, considering the lower proportions of participants who sought them out. Online self-guided therapies are recognized as a way to improve access to evidence-based mental health support [35], particularly in the face of overstretched public health systems. Their potential can only be realised if they are widely recognised as a viable option for mental health support. Developers of self-guided interventions should address known barriers to user engagement. These include users’ uncertainty about the evidence base for efficacy, as well as their perception that the interventions lack personalisation [36].

A second message for policymakers and mental health providers is about two specific sources: NHS phone lines and emergency mental health services. These two sources did not appear to meet participants’ expectations for support. Both NHS phone lines and emergency mental health services are intended for those seeking urgent care, who have the most serious and pressing needs. Adults seeking help from these sources may inherently present with the most difficult needs to address. A reason for treatment non-receipt for both sources was ‘I was assessed and they were unable to offer me support’. This suggests that referral onwards to an expected form of care or support was perceived as problematic. From our data, policymakers might look to examine the adequacy of treatment available to adults after they have been assessed.

A third finding with clinical implications relates to the demographic differences in treatment receipt in our data. For men and older adults, treatment receipt was markedly lower than for women and younger adults. There is a need to understand why men and older adults perceive a disproportionate treatment ‘gap’ and to address this gap. For older adults, there have been valuable suggestions in the literature on increasing access to mental health treatment, particularly related to digital mental health tools. These include designing for ease of use, accessibility, and providing technical skill training as part of the treatment’s delivery [11]. Targeting older adults’ trust and buy-in to digital tools and technologies [37], traditionally lower than for other age groups, may also be important in supporting uptake. For men, approaches aimed at increasing engagement and uptake of services in underserved populations may be required [38], such as co-design and user involvement in testing tools and programmes.

### Limitations

Our study used convenience sampling, and our cohort had an over-representation of women, adults reporting high levels of education and people reporting white ethnicity. These issues have been widely discussed as caveats of the COVID mental health literature [e.g., 39]. We used a set of questions about treatment seeking, co-designed with key stakeholders, but we do not have external data on actual service use (e.g., GP appointments) that would confirm our measure’s validity. Given the repeated assessment design of the RAMP and COPING studies where participants were asked about their recent experiences, we do not expect memory errors or recall biases to be as profound as studies asking participants to retrospectively report their treatment seeking behaviours over longer time frames. We also did not adjust for seasonal variation in population mental health [40], although others have estimated that the effects of seasonal variation are unlikely to account for changes in population mental health during the pandemic [41]. Another factor we did not adjust for was regional variation. There were local lockdowns implemented in response to COVID-19 case spikes that were limited to specific geographic areas. Additionally, there were minor differences in the timing of lockdowns in Northern Ireland, Wales, England and Scotland.

We prioritised asking participants about their experiences with a comprehensive range of mental health treatment and support options. However, we did not collect fine-grained detail about the sources accessed by participants. For example, we cannot describe which websites or online self-guided treatments participants accessed and there are well-described safety concerns about some consumer-facing health apps [42]. We also did not collect data on the duration or intensity of engagement with each source, for example, to understand whether participants briefly browsed a website or engaged for several weeks with a structured therapeutic activity such as mindfulness.

## Conclusions

Across 12 months of the pandemic in the UK, the majority (57%) of adults who reported seeking help in our sample received it. For our participants, online talking therapy was one of the most frequently sought out sources of help. In contrast, self-guided online therapy was one of the least frequently sought sources. Not only were online talking therapies frequently sought, but they were also rated typically as being extremely or very helpful to those who did access them. Our findings underline online talking therapies as an acceptable, widely sought source for mental health support, of importance when considering the current NHS waiting lists. Telephone lines, including those provided by both NHS and non–government services, had marked treatment ‘gaps’, particularly for men and older adults. We did not find substantial fluctuations in treatment-seeking over the study period, with most of our participants who did seek help looking for support for a mental health concern that was either continuing or worsening.

## Supporting information

S1 Table Proportion of individuals responding to questionnaires and seeking treatment across assessed time points. (DOCX)

S2 Table Rates of treatment seeking and receipt across sources of support. (DOCX)

S3 Table Rates of treatment seeking and receipt across sources of support, separated by gender. (DOCX)

S4 Table Rates of treatment seeking across sources of support, separated by age. (DOCX)

S5 Table Rates of treatment receipt across sources of support, separated by age. (DOCX)

S6 Table Reasons for seeking treatment across assessment intervals. (DOCX)

S7 Table Reasons for treatment non-receipt by sources of support. (DOCX)

S8 Table Reasons for treatment non-receipt by gender. (DOCX)

S9 Table Ratings of helpfulness across sources of support. (DOCX)
