# Supplementary material for: Seeking help for mental health during the COVID-19 pandemic: A longitudinal analysis of adults’ experiences with digital technologies and services
Source: PLOS Digit Health. 2023 Dec 6;2(12):e0000402. doi: 10.1371/journal.pdig.0000402 (PMC10699588; doi:10.1371/journal.pdig.0000402)
Supplement: S1 Table — (DOCX) [file pdig.0000402.s001.docx]

*Additional methodological details*

Questionnaires involved multiple-choice answers, individuals who did not report on key variables at each interval were included in analyses of that interval. No attention checks were included in the questionnaire schedule.

Table S1 shows the number and proportion of individuals responding to surveys and seeking treatment across intervals. In RAMP, questionnaires were sent monthly in July, August and September, then every two months for November 2020-June 2021. In COPING, questionnaires were sent every two months. Note that although questionnaires were sent at two-monthly intervals, participants could respond at later dates. Data was organised into intervals of a calendar month, based on the date of responding. Response rates were largely consistent for COPING and more variable for RAMP.

**Table S1.** Proportion of individuals responding to questionnaires and seeking treatment across assessed time points

| **Interval** | **N responses COPING** | **% of total COPING sample** | **N responses RAMP** | **% of total RAMP sample** | **Total responses** | **N seeking** | **% seeking treatment** | **% received** |
| --- | --- | --- | --- | --- | --- | --- | --- | --- |
| Jul ‘20 | 170 | 0.8 | 4249 | 77.2 | 4419 | 667 | 15.09 | 55.10 |
| Aug ‘20 | 14175 | 64.2 | 3675 | 66.7 | 17850 | 2398 | 13.43 | 56.81 |
| Sep ‘20 | 1156 | 5.2 | 3437 | 62.4 | 4593 | 737 | 16.05 | 59.62 |
| Oct ‘20 | 14608 | 66.2 | 97 | 1.8 | 14705 | 2005 | 13.63 | 53.88 |
| Nov ‘20 | 1335 | 6.0 | 3148 | 57.2 | 4483 | 762 | 17.00 | 54.61 |
| Dec ‘20 | 13652 | 61.9 | 211 | 3.8 | 13863 | 1743 | 12.57 | 59.49 |
| Jan ‘21 | 1650 | 7.5 | 2885 | 52.4 | 4535 | 681 | 15.02 | 52.58 |
| Feb ‘21 | 14325 | 64.9 | 36 | 0.7 | 14361 | 1838 | 12.80 | 59.92 |
| Mar ‘21 | 1113 | 5.0 | 2566 | 46.6 | 3679 | 583 | 15.85 | 56.94 |
| Apr ‘21 | 14497 | 65.7 | 12 | 0.2 | 14509 | 1901 | 13.10 | 58.96 |
| May ‘21 | 5 | 0.0 | 2030 | 36.9 | 2035 | 282 | 13.86 | 65.56 |
| Jun ‘21 | 13490 | 61.1 | 1267 | 23.0 | 14757 | 1857 | 12.58 | 57.86 |
