# Supplementary material for: Seeking help for mental health during the COVID-19 pandemic: A longitudinal analysis of adults’ experiences with digital technologies and services
Source: PLOS Digit Health. 2023 Dec 6;2(12):e0000402. doi: 10.1371/journal.pdig.0000402 (PMC10699588; doi:10.1371/journal.pdig.0000402)
Supplement: S2 Table — (DOCX) [file pdig.0000402.s002.docx]

**Table S2.** Rates of treatment seeking and receipt across sources of support.

| **Source of support** | **N seeking attempts** | **% Sought** | **% Received** | **% Not received** |
| --- | --- | --- | --- | --- |
| GP | 5106 | 19.94 | 59.93 | 40.07 |
| Existing MH team | 4071 | 15.90 | 62.74 | 37.26 |
| Online talk therapy | 3917 | 15.30 | 64.82 | 35.18 |
| Structured therapeutic activity | 2615 | 10.21 | 69.56 | 30.44 |
| Non-government website | 1854 | 7.24 | 48.33 | 51.67 |
| Other | 1808 | 7.06 | 57.91 | 42.09 |
| Government website | 1776 | 6.94 | 39.47 | 60.53 |
| Non-NHS phone line | 1356 | 5.30 | 53.61 | 46.39 |
| Online Self-guided | 1271 | 4.96 | 50.90 | 49.10 |
| Emergency MH team | 1275 | 4.98 | 37.73 | 62.27 |
| NHS phoneline (111) | 556 | 2.17 | 39.93 | 60.07 |
