# Supplementary material for: Seeking help for mental health during the COVID-19 pandemic: A longitudinal analysis of adults’ experiences with digital technologies and services
Source: PLOS Digit Health. 2023 Dec 6;2(12):e0000402. doi: 10.1371/journal.pdig.0000402 (PMC10699588; doi:10.1371/journal.pdig.0000402)
Supplement: S3 Table — (DOCX) [file pdig.0000402.s003.docx]

**Table S3.** Rates of treatment seeking and receipt across sources of support, separated by gender.

| **Source of support** | **% Sought** | | **% Received** | |
| --- | --- | --- | --- | --- |
|  | **Male** | **Female** | **Male** | **Female** |
| GP | 20.28 | 19.99 | 56.48 | 60.61 |
| Existing MH team | 17.13 | 15.63 | 57.16 | 63.54 |
| Online talk therapy | 13.21 | 15.65 | 60.60 | 65.17 |
| Structured therapeutic activity | 9.57 | 10.41 | 64.51 | 70.51 |
| Non-government website | 6.40 | 7.36 | 41.86 | 49.08 |
| Other | 6.47 | 7.23 | 56.32 | 57.56 |
| Government website | 6.17 | 7.02 | 36.95 | 39.56 |
| Non-NHS phone line | 5.06 | 5.30 | 40.20 | 55.68 |
| Online Self-guided | 6.02 | 4.75 | 42.39 | 52.76 |
| Emergency MH team | 6.87 | 4.62 | 33.94 | 38.59 |
| NHS phoneline (111) | 2.83 | 2.04 | 28.07 | 43.57 |
