# Supplementary material for: Seeking help for mental health during the COVID-19 pandemic: A longitudinal analysis of adults’ experiences with digital technologies and services
Source: PLOS Digit Health. 2023 Dec 6;2(12):e0000402. doi: 10.1371/journal.pdig.0000402 (PMC10699588; doi:10.1371/journal.pdig.0000402)
Supplement: S4 Table — (DOCX) [file pdig.0000402.s004.docx]

**Table S4.** Rates of treatment seeking across sources of support, separated by age.

| **Source of support** | **% Sought** | | | | | | |
| --- | --- | --- | --- | --- | --- | --- | --- |
|  | **16-25** | **26-35** | **36-45** | **46-55** | **56-65** | **66-75** | **76+** |
| GP | 17.93 | 18.04 | 19.45 | 20.69 | 21.97 | 25.66 | 26.04 |
| Existing MH team | 15.91 | 17.37 | 16.25 | 15.51 | 13.19 | 14.98 | 15.63 |
| Online talk therapy | 16.13 | 17.91 | 15.24 | 14.16 | 13.57 | 14.16 | 18.75 |
| Structured therapeutic activity | 9.88 | 10.59 | 10.55 | 10.28 | 10.10 | 9.06 | 8.33 |
| Non-government website | 7.85 | 7.47 | 7.47 | 7.11 | 6.89 | 4.22 | 1.04 |
| Other | 5.11 | 5.94 | 7.23 | 7.25 | 9.29 | 11.01 | 14.58 |
| Government website | 8.40 | 7.20 | 7.40 | 6.18 | 6.48 | 5.22 | 5.21 |
| Non-NHS phone line | 6.17 | 4.46 | 5.07 | 5.25 | 5.9 | 4.14 | 1.04 |
| Online Self-guided | 5.48 | 5.15 | 4.83 | 5.15 | 4.84 | 4.3 | 4.17 |
| Emergency MH team | 5.14 | 3.87 | 4.58 | 6.02 | 5.47 | 3.97 | 3.13 |
| NHS phoneline (111) | 2.00 | 2.01 | 1.92 | 2.40 | 2.30 | 2.73 | 2.08 |
