# Supplementary material for: Seeking help for mental health during the COVID-19 pandemic: A longitudinal analysis of adults’ experiences with digital technologies and services
Source: PLOS Digit Health. 2023 Dec 6;2(12):e0000402. doi: 10.1371/journal.pdig.0000402 (PMC10699588; doi:10.1371/journal.pdig.0000402)
Supplement: S5 Table — (DOCX) [file pdig.0000402.s005.docx]

**Table S5.** Rates of treatment receipt across sources of support, separated by age.

| **Source of support** | **% Received** | | | | | | |
| --- | --- | --- | --- | --- | --- | --- | --- |
|  | **16-25** | **26-35** | **36-45** | **46-55** | **56-65** | **66-75** | **76+** |
| **Overall** | **63.93** | **62.59** | **56.53** | **54.50** | **50.20** | **53.31** | **42.71** |
| GP | 64.97 | 66.59 | 58.38 | 59.29 | 53.57 | 58.71 | 36.00 |
| Existing MH team | 73.61 | 63.86 | 62.82 | 60.85 | 52.98 | 51.93 | 33.33 |
| Online talk therapy | 67.26 | 70.09 | 63.17 | 61.64 | 58.58 | 67.25 | 72.22 |
| Structured therapeutic activity | 76.30 | 75.30 | 67.08 | 66.55 | 62.41 | 63.79 | 75.00 |
| Non-government website | 57.09 | 52.38 | 45.38 | 46.87 | 36.03 | 45.10 | 100.00 |
| Other | 62.01 | 63.73 | 57.61 | 54.55 | 56.13 | 55.64 | 35.71 |
| Government website | 47.28 | 43.90 | 38.19 | 31.70 | 35.94 | 31.75 | 20.00 |
| Non-NHS phone line | 60.65 | 61.50 | 51.49 | 50.85 | 44.64 | 48.00 | 0.00 |
| Online Self-guided | 59.38 | 56.10 | 53.57 | 47.75 | 41.36 | 36.54 | 0.00 |
| Emergency MH team | 51.67 | 36.76 | 38.68 | 34.91 | 32.87 | 22.92 | 0.00 |
| NHS phoneline (111) | 45.71 | 52.08 | 47.19 | 31.11 | 31.87 | 24.24 | 50.00 |
