# Supplementary material for: Seeking help for mental health during the COVID-19 pandemic: A longitudinal analysis of adults’ experiences with digital technologies and services
Source: PLOS Digit Health. 2023 Dec 6;2(12):e0000402. doi: 10.1371/journal.pdig.0000402 (PMC10699588; doi:10.1371/journal.pdig.0000402)
Supplement: S6 Table — (DOCX) [file pdig.0000402.s006.docx]

**Table S6.** Reasons for seeking treatment across assessment intervals.

|  | **Reason for seeking treatment (%)** | | | | |
| --- | --- | --- | --- | --- | --- |
| **Interval** | Existing mental health problem | Mental health crisis | Newly emerging symptoms | Seeking new treatment | Other |
| June 2021 | 72.18 | 9.78 | 9.16 | 5.60 | 3.29 |
| May 2021 | 67.83 | 11.03 | 12.04 | 4.35 | 4.74 |
| April 2021 | 74.00 | 9.19 | 8.56 | 5.12 | 3.13 |
| March 2021 | 70.73 | 10.42 | 10.55 | 4.00 | 4.31 |
| February 2021 | 73.59 | 8.99 | 8.85 | 5.55 | 3.03 |
| January 2021 | 71.22 | 10.55 | 10.38 | 4.85 | 3.00 |
| December 2020 | 72.81 | 9.58 | 8.81 | 4.96 | 3.84 |
| November 2020 | 70.76 | 9.58 | 11.08 | 4.77 | 3.82 |
| October 2020 | 73.26 | 8.81 | 9.09 | 4.96 | 3.88 |
| September 2020 | 70.29 | 11.28 | 10.34 | 3.93 | 4.16 |
| August 2020 | 73.28 | 9.21 | 9.55 | 4.62 | 3.34 |
| July 2020 | 69.30 | 11.76 | 11.31 | 4.44 | 3.19 |
