# Supplementary material for: Seeking help for mental health during the COVID-19 pandemic: A longitudinal analysis of adults’ experiences with digital technologies and services
Source: PLOS Digit Health. 2023 Dec 6;2(12):e0000402. doi: 10.1371/journal.pdig.0000402 (PMC10699588; doi:10.1371/journal.pdig.0000402)
Supplement: S7 Table — (DOCX) [file pdig.0000402.s007.docx]

**Table S7.** Reasons for treatment non-receipt by sources of support.

| **Source of support** | **Reason** | | | | | | | | |
| --- | --- | --- | --- | --- | --- | --- | --- | --- | --- |
|  | Could not get an appointment | Waiting list was too long | Was assessed and was unable to be offered  support | None of the support options were relevant | Support option was unavailable when tried to  access it | Didn't feel well enough to engage | Was too busy to engage | Felt better | Other |
| GP | 27.24 | 6.95 | 13.29 | 9.06 | 7.52 | 5.10 | 1.70 | 4.74 | 24.41 |
| Existing MH team | 17.95 | 14.39 | 12.29 | 8.31 | 13.48 | 7.47 | 1.68 | 3.28 | 21.16 |
| Online talk therapy | 14.34 | 18.14 | 6.67 | 7.21 | 9.69 | 8.53 | 4.57 | 5.35 | 25.50 |
| Structured therapeutic activity | 5.01 | 5.01 | 1.81 | 9.74 | 6.40 | 24.62 | 9.87 | 11.96 | 25.59 |
| Non-government website | 3.80 | 6.15 | 2.91 | 23.27 | 9.51 | 19.46 | 4.03 | 6.94 | 23.94 |
| Other | 9.66 | 9.34 | 4.51 | 7.73 | 8.86 | 7.41 | 4.19 | 6.92 | 41.38 |
| Government website | 4.86 | 6.14 | 3.87 | 28.34 | 9.51 | 14.07 | 4.86 | 5.15 | 23.19 |
| Non-NHS phone line | 6.72 | 9.82 | 5.04 | 13.31 | 11.24 | 18.99 | 4.13 | 9.95 | 20.8 |
| Online Self-guided | 6.40 | 8.30 | 2.60 | 11.94 | 7.61 | 18.51 | 10.55 | 9.00 | 25.09 |
| Emergency MH team | 16.4 | 12.5 | 17.61 | 9.14 | 11.42 | 12.23 | 0.94 | 4.97 | 14.78 |
| NHS phoneline (111) | 6.51 | 6.84 | 20.52 | 15.64 | 10.10 | 12.05 | 1.30 | 9.45 | 17.59 |
