# Supplementary material for: Seeking help for mental health during the COVID-19 pandemic: A longitudinal analysis of adults’ experiences with digital technologies and services
Source: PLOS Digit Health. 2023 Dec 6;2(12):e0000402. doi: 10.1371/journal.pdig.0000402 (PMC10699588; doi:10.1371/journal.pdig.0000402)
Supplement: S8 Table — (DOCX) [file pdig.0000402.s008.docx]

**Table S8.** Reasons for treatment non-receipt by gender.

| **Reason for non-receipt** | **Male (%)** | **Female (%)** |
| --- | --- | --- |
| Could not get an appointment | 12.51 | 13.65 |
| Waiting list was too long | 8.58 | 9.86 |
| Was assessed and was unable to be offered support | 9.57 | 8.28 |
| None of the support options were relevant | 12.56 | 12.56 |
| Support option was unavailable when tried to access it | 11.01 | 9.33 |
| Didn't feel well enough to engage | 12.17 | 12.08 |
| Was too busy to engage | 2.77 | 4.18 |
| Felt better | 9.24 | 5.79 |
| Other | 21.58 | 24.27 |
