# Supplementary material for: Seeking help for mental health during the COVID-19 pandemic: A longitudinal analysis of adults’ experiences with digital technologies and services
Source: PLOS Digit Health. 2023 Dec 6;2(12):e0000402. doi: 10.1371/journal.pdig.0000402 (PMC10699588; doi:10.1371/journal.pdig.0000402)
Supplement: S9 Table — (DOCX) [file pdig.0000402.s009.docx]

**Table S9.** Ratings of helpfulness across sources of support.

| **Source of support** | **Helpfulness rating (%)** | | | | | | |
| --- | --- | --- | --- | --- | --- | --- | --- |
|  | **Extremely**  **unhelpful** | **Very**  **unhelpful** | **Somewhat**  **unhelpful** | **Neither**  **helpful nor unhelpful** | **Somewhat**  **helpful** | **Very**  **helpful** | **Extremely**  **helpful** |
| GP | 2.29 | 2.82 | 5.63 | 6.39 | 35.30 | 27.87 | 19.71 |
| Existing MH team | 1.96 | 3.06 | 5.93 | 6.83 | 31.83 | 28.02 | 22.37 |
| Online talk therapy | 2.57 | 2.33 | 4.07 | 7.47 | 29.96 | 28.93 | 24.66 |
| Structured therapeutic  activity | 1.43 | 2.09 | 3.58 | 7.21 | 44.60 | 27.42 | 13.66 |
| Non-government website | 1.35 | 1.57 | 4.26 | 10.76 | 45.18 | 25.22 | 11.66 |
| Other | 1.93 | 2.80 | 3.38 | 7.63 | 30.82 | 31.88 | 21.55 |
| Government website | 1.58 | 1.72 | 5.60 | 15.64 | 53.23 | 19.08 | 3.16 |
| Non-NHS phone line | 2.76 | 2.76 | 7.60 | 7.87 | 38.67 | 25.41 | 14.92 |
| Online Self-guided | 2.48 | 2.64 | 6.05 | 13.49 | 45.27 | 20.16 | 9.92 |
| Emergency MH team | 3.56 | 4.40 | 15.09 | 11.53 | 32.49 | 19.08 | 13.84 |
| NHS phoneline (111) | 2.26 | 3.17 | 8.14 | 8.60 | 34.84 | 29.41 | 13.57 |
